# Commentary: Systemic Lupus Erythematosus in Native sub-Saharan Africans: A Systematic Review and Meta-Analysis

**DOI:** 10.3389/fmed.2020.00202

**Published:** 2020-05-27

**Authors:** Sandro Vento, Francesca Cainelli

**Affiliations:** ^1^Faculty of Medicine, University of Puthisastra, Phnom Penh, Cambodia; ^2^Raffles Medical Group Clinic, Phnom Penh, Cambodia

**Keywords:** systemic lupus erythematosus, autoimmune diseases, sub-Saharan Africa, epidemiology, rheumatology

Low and middle-income countries are thought to have a high frequency of infectious diseases and a low rate of autoimmune and allergic diseases (1); this belief has supported the “hygiene hypothesis,” i.e., the idea that a decreased incidence of infections contributes directly to an increased frequency of autoimmune and allergic diseases worldwide (2). In particular, the incidence of systemic lupus erythematosus (SLE) in black Africans has long been considered negligible (3). A recent systematic review and meta-analysis on SLE in native sub-Saharan Africans (4) challenges this view and changes the perspective. By examining studies published between January 2008 and October 2018 on SLE in native sub-Saharan Black Africans, the pooled prevalence of the disease among 28,575 participants in Internal Medicine and Rheumatology Units was found to be 1.7%. Patients were diagnosed between 1987 and 2014, and the mean age at diagnosis was 28.8–39.2 years (4). Studies from only 11 countries, the majority of which located in West Africa, were included in the analysis, and the pooled mortality rate was 10.3%, with infections, kidney disease, neurological involvement, and SLE flares as the main causes (4). The patients had 89.7% prevalence rate for antinuclear antibodies and 54.6% for anti-DNA antibodies, and a high seroprevalence for anti-ribonucleoprotein (57.9%), anti-Smith (53.5%), anti-Sjogren syndrome antigen A (45.6%) and anti-Sjogren syndrome antigen B (33.7%) autoantibodies (4). The high seroprevalence rates of ENA autoantibodies confirms their higher frequency in Blacks, due to genetic susceptibility (5).

The authors outlined that the prevalence from their study contrasted with the low number of SLE cases described by Bae et al. (3) over 20 years before, in spite of comparable data sources and geographical coverage.

Importantly, a review of published studies is different from a review of all cases diagnosed with SLE in all hospitals or in referral hospitals of certain countries over a defined period of time, and is likely to underestimate the real number of observed hospital cases.

Three questions arise from the results of this review, that estimated a hospital-based prevalence in urban areas: is the rise in diagnoses due to improved diagnostic capacity of sub-Saharan African doctors, or to a real increase in the number of cases? What is the prevalence of SLE in the general population? Are other autoimmune diseases also more frequent than commonly thought?

It is very difficult to answer the first question; while the fact that the number of rheumatologists is still very low in sub-Saharan Africa [even South Africa has an estimated ratio of only one rheumatologist for every 820,000 inhabitants (6), and in Ghana two specialists serve the whole country (7)] argues against an improved diagnostic capacity, continuing urbanization (Africa is the fastest urbanizing continent) and consequent proximity to referral hospitals of a higher number of potential patients may explain the increase in diagnoses. Registries and cohorts are needed to better establish the epidemiology of SLE in sub-Saharan Africa; the African Lupus Genetics Network (ALUGEN) registry (8) is an important initiative that will hopefully allow to gather comprehensive, multi-ethnic data on African SLE patients, and to establish whether the frequency of the disease differs between urban and rural areas. It would be particularly interesting to compare the prevalence of SLE in members of similar tribal groups living in their traditional homeland with the prevalence in those who have moved to cities to clarify the possible importance of environmental factors in the occurrence of the disease.

Other autoimmune diseases are diagnosed with increasing frequency in sub-Saharan Africa (9, 10); interestingly, the prevalence estimates of rheumatoid arthritis in Africa (0.36%), according to two systematic analyses published in 2012 (11) and 2015 (12), are comparable with those in Southern Europe (0.33%), even though lower than the estimates for Northern Europe (0.50%), and USA (1.07%) (13).

Extensive under–reporting was noticed in hospital–based vs. population-based studies for rheumatoid arthritis in sub-Saharan Africa (11), with a 6- to 10-fold reduction in hospital reports. Should this hold true for SLE, its prevalence in the sub-continent could be considerably higher than the one that can be inferred from the results of Essouma's et al. review. In fact, milder cases of SLE may not present to hospital or be treated as anemia with skin rash by general physicians.

If the occurrence of SLE and other autoimmune diseases is not, or no longer, negligible in sub-Saharan Africa, a huge effort will have to be made to give the patients the care they deserve. First, many more rheumatologists will have to be trained, and medical schools around the continent must consider this need. Mortality in hospitals that treat larger numbers of SLE patients is lower than expected, and the presence of specialists in the main referral hospitals could allow the creation of referral centers in every country. Second, autoantibody detection will have to performed at least in the main referral hospitals of each country; currently these expensive diagnostic tests are very often not available or unaffordable for the patients, and samples are sent to accredited laboratories in South African or European countries, with a lengthy turn-around time, and a huge cost (the cost of a complete set of serological tests for SLE can be as high as 500 USD). Third, more pathologists will have to be trained; they are presently too few, and histopathological reports of kidney biopsies, so important in diagnosing and staging of lupus nephritis that often occurs in Black patients, take often far too long to reach the physician. Fourth, attention will have to be made to acute infections and tuberculosis, that occurs frequently in SLE patients in the continent, and dialysis will need to be available for patients with lupus nephritis.

SLE can no longer be neglected in sub-Saharan Africa; the patients deserve access to much better and affordable standards of care in the whole continent and it is time to plan and provide those.

## Author Contributions

SV and FC wrote the Commentary and contributed equally to it.

## Conflict of Interest

The authors declare that the research was conducted in the absence of any commercial or financial relationships that could be construed as a potential conflict of interest.
